# Safety and Immunogenicity of Respiratory Syncytial Virus Prefusion Maternal Vaccine Coadministered With Diphtheria-Tetanus-Pertussis Vaccine: A Phase 2 Study

**DOI:** 10.1093/infdis/jiad560

**Published:** 2023-12-22

**Authors:** Nerea Hermida, Murdo Ferguson, Isabel Leroux-Roels, Sandra Pagnussat, Deborah Yaplee, Nancy Hua, Peter van den Steen, Bruno Anspach, Ilse Dieussaert, Joon Hyung Kim

**Affiliations:** Clinical Research Development, GSK Vaccines, Wavre, Belgium; Department of Family Medicine and Emergency Medicine, Colchester Research Group, Truro, Nova Scotia, Canada; Center for Vaccinology, Ghent University and Ghent University Hospital, Ghent, Belgium; Miami Research Associates, Miami, Florida, USA; Vaccine Development, GSK Vaccines, Rockville, Maryland, USA; Vaccine Development, GSK Vaccines, Rockville, Maryland, USA; Clinical Research Development, GSK Vaccines, Wavre, Belgium; Vaccine Development, GSK Vaccines, Rockville, Maryland, USA; Clinical Research Development, GSK Vaccines, Wavre, Belgium; Vaccine Development, GSK Vaccines, Rockville, Maryland, USA

**Keywords:** respiratory syncytial virus, maternal vaccine, RSV vaccine, safety, immunogenicity, coadministration, dTpa vaccine

## Abstract

**Background:**

Respiratory syncytial virus (RSV) fusion protein stabilized in the prefusion conformation (RSVPreF3) was under investigation as a maternal vaccine.

**Methods:**

This phase 2, randomized, placebo-controlled, single-dose, multicenter study enrolled healthy, nonpregnant women, randomized 1:1:1:1:1 to 5 parallel groups studying RSVPreF3 (60 or 120 µg) coadministered with diphtheria, tetanus, and acellular pertussis vaccine (dTpa) or placebo, and dTpa coadministered with placebo. Safety and humoral immune responses were assessed. An extension phase also assessed a RSVPreF3 120 μg vaccination 12–18 months after first vaccination.

**Results:**

The safety profile of RSVPreF3 was unaffected by dose or dTpa coadministration. Solicited and unsolicited adverse events (AEs) were evenly distributed across study groups. Injection-site pain was higher following the second vaccination versus the first vaccination. Medically attended AEs were rare (<5% overall). Both RSVPreF3 dose levels (alone and with dTpa) were immunogenic, increasing levels of RSV-A neutralizing antibody ≥8-fold and anti-RSVPreF3 IgG antibody ≥11-fold at 1 month postvaccination, which persisted at 12–18 months postvaccination; modest 2-fold increases were observed with a second RSVPreF3 vaccination.

**Conclusions:**

This study indicates RSVPreF3 coadministration with dTpa induces robust immune responses and is well tolerated, regardless of the RSVPreF3 dose level used.

**Clinical Trials Registration:**

NCT04138056.

## Teaser

This respiratory syncytial virus (RSV) fusion protein stabilized in the prefusion conformation (RSVPreF3) vaccine was tolerable when coadministered with diphtheria, tetanus, and acellular pertussis vaccine (dTpa) in healthy nonpregnant women, and induced robust immune responses.

Respiratory syncytial virus (RSV) is the leading cause of viral lower respiratory tract infection (LRTI) worldwide, with 50%–70% of newborns becoming infected during their first winter [1, 2]. Although RSV infections can occur at any age, infants have the highest incidence of severe disease (including bronchiolitis and pneumonia), with hospitalization peaking at around 1 to 3 months [3–5]. Furthermore, RSV-induced LRTI is the most common cause of hospitalization among infants <6 months of age in the United States [6]. An effective maternal RSV vaccine could have a substantial effect on reducing disease burden in this age group [7, 8].

RSV fusion (F) protein stabilized in the prefusion conformation (RSVPreF3) was an investigational, intramuscular, RSV maternal vaccine aiming to prevent RSV-associated illness in infants via maternal antibody transfer. RSVPreF3 was intended for administration as a single dose in the late second or third trimester of pregnancy; therefore, it was important to evaluate any potential interference of coadministration with diphtheria, tetanus, and acellular pertussis (dTpa) vaccine (Boostrix; GSK), which is currently recommended for maternal vaccination in more than 40 countries worldwide [9–11]. Depending on the country, dTpa vaccination is recommended in either the late second or third trimester to protect newborns in the first months of life, when they are too young to be vaccinated [11, 12].

Following a previous phase 1/2 clinical trial (NCT03674177) that investigated RSVPreF3 doses of 30, 60, and 120 μg [13], 2 dose levels (60 and 120 μg) were selected for further evaluation. Herein, we present results from a phase 2 study evaluating the safety and immunogenicity of RSVPreF3 and dTpa, when given alone or coadministered, in healthy, nonpregnant women. Two formulations of dTpa, containing either 300 μg (dTpa_300, licensed in the United States) or 500 μg (dTpa_500, licensed outside the United States; ex-US) of aluminum salts, were used. In an extension phase, persistence of the response to RSVPreF3 was measured from 12 to 18 months following the first vaccination. A second vaccination, with RSVPreF3 (120 μg), was administered between 12 and 18 months after the first vaccination, to evaluate safety and immunogenicity, and to determine if additional dosing might be required for women with successive pregnancies.

## METHODS

### Study Design

**Figure 1.**
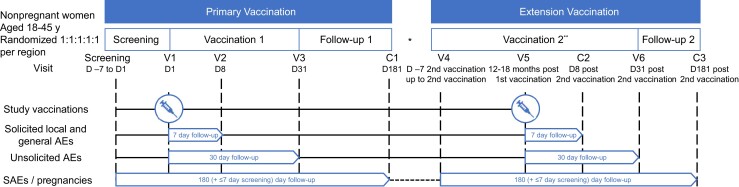
Study design. Abbreviations: AE, adverse event; C, phone contact; D, day; RSV, respiratory syncytial virus; SAE, serious adverse event; V, visit. *There was no follow-up between contact 1 and visit 4. **All participants received RSV 120 μg and were followed in an unblinded, open-label manner.

This phase 2, randomized, placebo-controlled, self-contained, multicenter study was conducted between 5 November 2019 and 22 November 2021, at 8 centers within Belgium (2 centers), Canada (2 centers), and the United States (4 centers). It was conducted in an observer-blinded manner until 6 months after the first vaccination. Participants were randomized 1:1:1:1:1 to the following groups: 60 μg of RSVPreF3 (GSK388550A) and dTpa (RSV60_dTpa); 120 μg of RSVPreF3 and dTpa (RSV120_dTpa); 60 μg of RSVPreF3 and placebo (RSV60_placebo); 120 μg of RSVPreF3 and placebo (RSV120_placebo); or dTpa and placebo (dTpa_placebo). Full randomization details are available in the Supplementary Materials. Planned enrollment was approximately 500 participants (250 in the United States receiving dTpa_300, and 250 outside United States receiving dTpa_500). During the primary phase, participants were followed-up for approximately 6 months (Figure 1). Instituted in a protocol amendment, the extension phase started 12 to 18 months after the initial vaccination, with participants receiving an additional RSVPrF3 administration and 6 months further follow-up (Figure 1). Details on vaccine composition, dosage, and route of administration are provided in the Supplementary Materials.

The study was registered on ClinicalTrials.gov (NCT04138056), and conducted in accordance with the Declaration of Helsinki, the principles of Good Clinical Practice, and all applicable regulatory requirements.

### Participants

The primary phase enrolled healthy, nonpregnant women (determined by medical history and clinical examination), aged 18–45 years at the time of first vaccination, who provided written informed consent. Participants who completed the primary vaccination phase, fulfilled all eligibility criteria, and provided written informed consent were enrolled into the extension phase. Further eligibility criteria are provided in the Supplementary Materials.

### Objectives

The primary safety objectives were to evaluate safety of RSVPreF3 alone or coadministered with dTpa, from first vaccination to day 31 after first vaccination and from second vaccination to day 31 after second vaccination. The primary immunogenicity objective was to evaluate the humoral response (RSV-A neutralizing antibody [RSV-A nAb] titers, and RSV immunoglobulin G [IgG] antibody concentrations) to RSVPreF3 alone or coadministered with dTpa, from first vaccination to day 31. Secondary safety objectives included to evaluate pooled safety across all RSVPreF3 groups and safety up to day 181 after second vaccination. Secondary immunogenicity objectives included to evaluate humoral response (RSV-A nAb titers, and RSV IgG antibody concentrations) at 12–18 months after first vaccination; humoral response to pertussis, diphtheria, and tetanus components at screening and day 31 after first vaccination; and humoral response after second vaccination. Objectives and end points are fully summarized in Supplementary Table 1.

### Assessments

#### Safety Assessments

Solicited local (pain, redness, swelling) and general (fatigue, fever, gastrointestinal symptoms, headache) adverse events (AEs) were recorded on diary cards for 7 days postvaccination. Unsolicited AEs were collected up to day 31 (30 days postvaccination). AE intensity was graded 0 (none/normal [used for solicited AEs only]); 1 (mild); 2 (moderate); or 3 (severe). All serious AEs (SAEs) were followed until event resolution, stabilization, they were otherwise explained, or subject was lost to follow-up. All solicited local AEs were considered causally related to vaccination, and causality of all other AEs was assessed by the investigator.

#### Immunological Assessment

Blood samples were collected at screening, days 8 and 31 after first vaccination, as well as prior to the second vaccination and 31 days after second vaccination. Anti-RSV-A nAbs and anti-RSVPreF3 IgG antibodies were measured using an in-house enzyme linked immunosorbent assay (ELISA) described previously [13]. Anti-RSV-A nAb titers were expressed as the estimated dilution 60 (ED_60_), corresponding to the inverse of the interpolated serum dilution that yields a 60% reduction in the number of plaques compared to the virus control wells; the assay cutoff was 18 ED_60_ [13]. Anti-RSVPreF3 IgG assay cutoff was 25 ELISA units/mL. Antibodies against diphtheria toxin (anti-D), tetanus toxin (anti-T), and the 3 pertussis antigens (anti-pertussis toxoid [PT], anti-filamentous hemagglutinin [FHA], and anti-pertactin [PRN]) were measured in house at GSK, by ELISA, with seropositivity cutoffs of 0.030, 0.037, 2.693, 2.046, and 2.187 IU/mL, respectively. Cellular immunity was not investigated.

### Statistical Analyses

#### Safety Analyses

The primary safety analyses were performed on the exposed set (all participants who received ≥1 dose of study treatment) and solicited safety set (exposed set participants who had solicited safety data). Analyses were performed on the pooled dTpa formulations and the US and ex-US dTpa formulations separately. The percentage of participants reporting solicited and unsolicited AEs were tabulated with exact 95% confidence intervals (CIs). The same computations were performed for grade 3 AEs, SAEs, vaccine-related AEs (all and grade 3), and medically attended AEs. Unsolicited AEs and SAEs were classified according to the Medical Dictionary for Regulatory Activities version 25.1. The percentage of participants using concomitant medication up to day 31 was summarized and pooled. Secondary analyses are summarized in the Supplementary Materials.

#### Immunogenicity Analyses

The primary immunogenicity analysis was based on the per protocol set (all participants who received ≥1 dose of the study treatment to which they were randomized, and had postvaccination data, with no deviations leading to study exclusion). Geometric mean titers (GMTs) and geometric mean concentrations (GMCs) of RSV-A nAb and RSV IgG, and their ratios (pre-/postvaccination time point) were tabulated with 95% CIs. Statistical analyses were performed using SAS 9.4 (Supplementary Materials).

Unless otherwise stated, results are shown for analyses in which data for the dTpa formulations (dTpa_300 and dTpa_500) were pooled. Results for analyses conducted separately for each formulation (US and ex-US dTpa formulations) were found to be similar to those shown for the pooled dataset. The patterns and magnitude of immune response to RSVPreF3 following the first and second vaccinations were similar when dTpa data were analyzed separately, and when data were pooled for the 2 different dTpa formulations.

## RESULTS

### Participant Disposition

**Figure 2.**
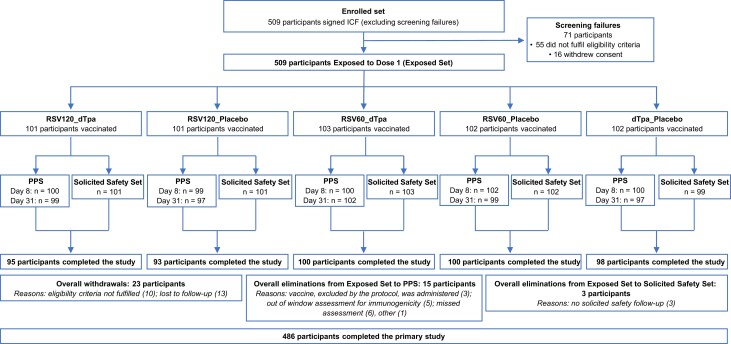
Consolidated Standards of Reporting Trials (CONSORT) flow chart of participant enrollment, group allocation, and elimination/exclusions in the primary phase. Abbreviations: dTpa, diphtheria, tetanus, and acellular pertussis; dTpa_placebo, participants who received dTpa and placebo; ICF, informed consent form; PPS, per protocol set; RSV, respiratory syncytial virus; RSV60_dTpa, participants who received RSV60 and dTpa; RSV60_placebo, participants who received RSV60 and placebo; RSV120_dTpa, participants who received RSV120 and dTpa; RSV120_placebo, participants who received RSV120 and placebo.

In the primary phase, 509 participants were randomized and vaccinated with the first dose of the study intervention, and 486 participants (95.5%) completed the phase (Figure 2). In the extension phase, 213 participants were vaccinated with RSVPreF3 (120 μg), as a second vaccination, and 208 participants (97.7%) completed the phase (Supplementary Figure 1).

### Demographic and Baseline Characteristics

The demographic characteristics in the exposed set were comparable across study groups and between the primary and extension phases (Table 1 and Supplementary Table 2). Overall, in the primary phase, mean age was 30.8 years and most participants were white (89.2%).

**Table 1.** Summary of Participant Characteristics Pooled for dTpa Formulation (Primary Phase)—Exposed Set

Characteristic	RSV120_dTpa (n = 101)	RSV120_Placebo (n = 101)	RSV60_dTpa (n = 103)	RSV60_Placebo (n = 102)	dTpa_Placebo (n = 102)	Total (n = 509)
Age at vaccination, y, mean (SD)	31.4 (7.6)	31.0 (8.0)	30.8 (8.4)	30.5 (8.3)	30.4 (7.9)	30.8 (8.0)
Age category at vaccination, y, No. (%)						
18–32	56 (55.4)	56 (55.4)	59 (57.3)	58 (56.9)	58 (56.9)	287 (56.4)
33–45	45 (44.6)	45 (44.6)	44 (42.7)	44 (43.1)	44 (43.1)	222 (43.6)
Country, No. (%)						
Belgium	29 (28.7)	29 (28.7)	30 (29.1)	29 (28.4)	29 (28.4)	146 (28.7)
Canada	21 (20.8)	20 (19.8)	21 (20.4)	22 (21.6)	22 (21.6)	106 (20.8)
United States	51 (50.5)	52 (51.5)	52 (50.5)	51 (50.0)	51 (50.0)	257 (50.5)
Ethnicity, No. (%)						
Hispanic or Latinx	11 (10.9)	10 (9.9)	3 (2.9)	11 (10.8)	9 (8.8)	44 (8.6)
Not Hispanic or Latinx	90 (89.1)	91 (90.1)	100 (97.1)	91 (89.2)	93 (91.2)	465 (91.4)
Race, No. (%)						
American Indian or Alaska Native	3 (3.0)	0 (0.0)	1 (1.0)	1 (1.0)	0 (0.0)	5 (1.0)
Asian	1 (1.0)	3 (3.0)	2 (1.9)	4 (3.9)	4 (3.9)	14 (2.8)
Black or African American	4 (4.0)	6 (5.9)	8 (7.8)	3 (2.9)	4 (3.9)	25 (4.9)
Native Hawaiian or other Pacific Islander	0 (0.0)	0 (0.0)	0 (0.0)	0 (0.0)	0 (0.0)	0 (0.0)
White	91 (90.1)	90 (89.1)	90 (87.4)	92 (90.2)	91 (89.2)	454 (89.2)
Other	2 (2.0)	2 (2.0)	2 (1.9)	2 (2.0)	3 (2.9)	11 (2.2)
BMI, kg/m^2^, mean (SD)	26.8 (5.3)	26.7 (5.2)	26.9 (5.5)	26.2 (5.7)	26.9 (5.5)	26.7 (5.4)

Abbreviations: BMI, body mass index; dTpa, diphtheria, tetanus, and acellular pertussis; dTpa_placebo, participants who received dTpa and placebo; RSV, respiratory syncytial virus; RSV60_dTpa, participants who received RSV60 and dTpa; RSV60_placebo, participants who received RSV60 and placebo; RSV120_dTpa, participants who received RSV120 and dTpa; RSV120_placebo, participants who received RSV120 and placebo.

### Safety Outcomes

#### Primary Phase

##### Solicited Local Adverse Events

**Figure 3.**
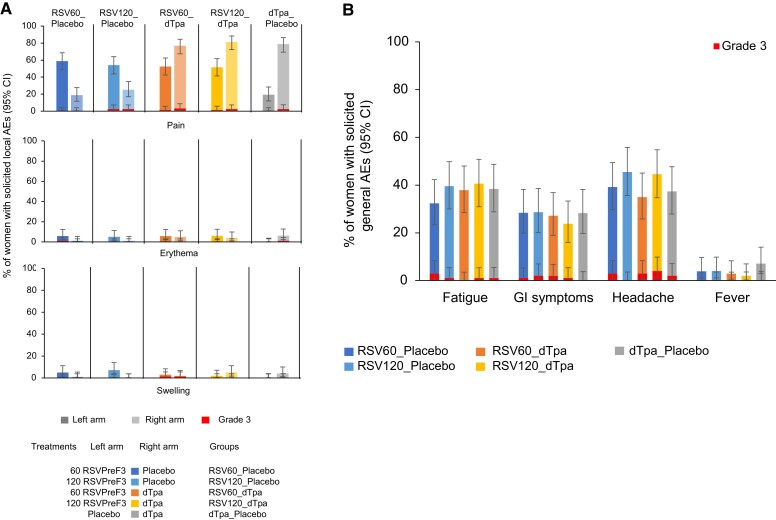
Solicited local AEs within 7 days of the first vaccination (*A*), and solicited general AEs within 7 days of the first vaccination (*B*), primary phase—solicited safety set. Abbreviations: AE, adverse event; CI, confidence interval; dTpa, diphtheria, tetanus, and acellular pertussis; dTpa_placebo, participants who received dTpa and placebo; GI, gastrointestinal; RSV, respiratory syncytial virus; RSV60_dTpa, participants who received RSV60 and dTpa; RSV60_placebo, participants who received RSV60 and placebo; RSV120_dTpa, participants who received RSV120 and dTpa; RSV120_placebo, participants who received RSV120 and placebo; RSVPreF3, RSV fusion protein stabilized in the prefusion conformation.

Frequency and severity of solicited local AEs were unaffected by coadministration and were found to be higher at the dTpa injection site compared with RSVPreF3. The most frequent solicited local AE in all study groups was injection-site pain, ranging from 51.5% to 58.8% for RSVPreF3 and 76.7% to 81.2% for dTpa (Figure 3*A* and Supplementary Table 3). The mean duration of solicited injection-site pain was ≤2.7 days and ≤3.3 days at the RSVPreF3 and dTpa injection sites, respectively. No medically attended solicited local AEs were reported. The frequency of participants reporting solicited grade 3 injection site pain, erythema, or swelling was ≤2% and ≤2.9% at the injection sites of RSVPreF3 and dTpa, respectively At the RSVPreF3 injection site, this included 4 cases of grade 3 pain, and 1 grade 3 case each of erythema and swelling (Supplementary Table 3). At the dTpa injection site, this included 9 cases of grade 3 pain, and 1 grade 3 case each of erythema and swelling.

##### Solicited General Adverse Events

Frequency and severity of solicited general AEs were similar when RSVPreF3 was administered with either dTpa or placebo. The most frequently reported solicited general AEs were headache (35.0%–45.5% across RSVPreF3 groups) and fatigue (32.4%–40.6% across RSVPreF3 groups) (Figure 3*B* and Supplementary Table 3). The mean duration of solicited general AEs was ≤2.6 days.

Fever (temperature ≥38.0°C) was infrequent: ≤ 4.0% (no grade 3 fever [≥ 39.0°C]) of participants (n = 4 in the RSV120_placebo group) reported fever across groups, and no cases were judged to be RSVPreF3 related. The frequencies of participants reporting grade 3 solicited general AEs were ≤2.9% (fatigue), ≤ 2.0% (gastrointestinal symptoms), and ≤4.0 (headache) (Supplementary Table 3). Medically attended solicited general AEs were infrequent with 1 participant (1.0%) in the RSV120_dTpa group reporting medically attended fever (>38.5 to ≤39°C) and 1 participant (1.0%) in each of the RSV120_dTpa and RSV60_dTpa groups reporting medically attended gastrointestinal symptoms. The proportion of patients across RSVPreF3 study groups with solicited general AEs related to RSVPreF3 was 29.7% for fatigue, 29.5% for headache, and 19.7% for gastrointestinal disorders.

##### Unsolicited Adverse Events

Unsolicited AEs were evenly distributed across study groups, ranging from 32.4% (dTpa_placebo) to 38.6% (RSV120_dTpa). The most common events were upper respiratory tract infection (5.3%; n = 27), headache (4.9%; n = 25), nasopharyngitis (2.4%; n = 12), and myalgia (2.2%; n = 11 (Table 2 and Supplementary Figure 2).

**Table 2.** Most Common Adverse Events Observed Within 30 Days of the First Vaccination (Reported in ≥3% of Patients in Any Treatment Group; Primary Phase)—Exposed Set

Adverse Event	RSV120_dTpa (n = 101)	RSV120_Placebo (n = 101)	RSV60_dTpa (n = 103)	RSV60_Placebo (n = 102)	dTpa_Placebo (n = 102)
Headache	8 (7.9) [3.5–15.0]	5 (5.0) [1.6–11.2]	6 (5.8) [2.2–12.2]	4 (3.9) [1.1–9.7]	2 (2.0) [.2–6.9]
Upper respiratory tract infection	8 (7.9) [3.5–15.0]	8 (7.9) [3.5–15.0]	4 (3.9) [1.1–9.6]	2 (2.0) [.2–6.9]	5 (4.9) [1.6–11.1]
Nasopharyngitis	1 (1.0) [.0–5.4]	1 (1.0) [.0–5.4]	0 (0) [.0–3.5]	7 (6.9) [2.8–13.6]	3 (2.9) [.6–8.4]
Myalgia	2 (2.0) [.2–7.0]	4 (4.0) [1.1–9.8]	1 (1.0) [.0–5.3]	4 (3.9) [1.1–9.7]	0 (0) [.0–3.6]
Injection-site bruising	1 (1.0) [.0–5.4]	0 (0) [.0–3.6]	4 (3.9) [1.1–9.6]	1 (1.0) [.0–5.3]	2 (2.0) [.2–6.9]
Administration-site erythema	0 (0) [.0–3.6]	2 (2.0) [.2–7.0]	4 (3.9) [1.1–9.6]	1 (1.0) [.0–5.3]	0 (0) [.0–3.6]
Fatigue	2 (2.0) [.2–7.0]	3 (3.0) [.6–8.4]	1 (1.0) [.0–5.3]	2 (2.0) [.2–6.9]	1 (1.0) [.0–5.3]
Oropharyngeal pain	3 (3.0) [.6–8.4]	2 (2.0) [.2–7.0]	1 (1.0) [.0–5.3]	1 (1.0) [.0–5.3]	0 (0) [.0–3.6]

Data are No. (%) [95% confidence interval].

Abbreviations: dTpa, diphtheria, tetanus, and acellular pertussis; dTpa_placebo, participants who received dTpa and placebo; RSV, respiratory syncytial virus; RSV60_dTpa, participants who received RSV60 and dTpa; RSV60_placebo, participants who received RSV60 and placebo; RSV120_dTpa, participants who received RSV120 and dTpa; RSV120_placebo, participants who received RSV120 and placebo.

The most common vaccine-related unsolicited AEs were myalgia (1.8%; n = 9), injection-site bruising (1.6%; n = 8), and injection-site pruritus (1.2%; n = 6). Grade 3 unsolicited AEs were reported by 15 participants (2.9%) across the study groups, with upper respiratory tract infection, gastroenteritis, gastrointestinal disorder, fatigue, and myalgia (0.4%; n = 2 each) the most common events. Four grade 3 unsolicited AEs considered related to vaccination were reported: myalgia (RSV120_dTpa, n = 1; RSV60_placebo, n = 1); chills and insomnia (both RSV120_placebo, n = 1). Medically attended AEs were reported by 24 participants (4.7%) across the study groups, ranging from 2.9% (RSV60_placebo group, n = 3) to 6.8% (RSV60_dTpa group, n = 7); the most frequent events were urinary tract infection (0.6%; n = 3), sinusitis (0.4%; n = 2), upper respiratory tract infection (0.4%; n = 2), and toothache (0.4%; n = 2).

#### Extension Phase

##### Solicited Local Adverse Events

Frequency and severity of solicited local AEs following RSVPreF3 120 μg as a second vaccine were similar in groups that had received RSVPreF3 in the primary phase. However, the frequency of injection-site pain was reported to be higher with the second vaccination and was the most frequently reported solicited local AE, ranging from 80.4% (RSV60_dTpa_RSV120 [95% CI, 66.1%–90.6%]) to 87.2% (RSV120_dTpa_RSV120 [95% CI, 72.6%–95.7%] and RSV120_placebo_RSV120 [95% CI, 72.6%–95.7%]). Only 38.6% (95% CI, 24.4%–54.5%) of participants in the study control group receiving RSVPreF3 for the first time (dTpa_placebo_RSV120) reported injection-site pain. The mean duration of solicited injection-site pain was ≤2.8 days in any study group. For each solicited local AE, ≤ 4.3% of participants in any study group reported a grade 3 event, consisting of 3 participants with erythema, and 1 participant each with pain and swelling.

##### Solicited General Adverse Events

Solicited general AEs were also similar in frequency and severity following a second vaccination with RSVPreF3. The most common events were headache (28.2% for RSV120_dTpa_RSV120; 56.1% for RSV60_placebo_RSV120) and fatigue (25.6% for RSV120_dTpa_RSV120; 46.3% for RSV60_placebo_RSV120). In the control group (dTpa_placebo_RSV120) receiving RSVPreF3 120 μg for the first time, 31.8% reported headache and 34.1% reported fatigue (Supplementary Table 4). The mean duration of each solicited general AE was ≤2.9 days in any study group.

Fever was rare, occurring in ≤4.9% of participants (RSV60_placebo_RSV120) with no RSVPreF3-related cases. One participant (2.4%) reported grade 3 fever in the RSV60_placebo_RSV120 group. Four participants reported a grade 3 solicited general AE. There was 1 case (2.4%) of medically attended event (fever >39.0 to ≤39.5°C) in the RSV60_placebo_RSV120 group. The proportion of patients across RSVPreF3 study groups with solicited general AEs related to RSVPreF3 was 32.1% for fatigue, 31.1% for headache, and 14.8% for gastrointestinal disorders.

##### Unsolicited Adverse Events

See Supplementary Materials.

### Immunogenicity Outcomes

#### Effect of dTpa Coadministration on Humoral Immune Responses to RSVPreF3-Related Antigens

##### Primary Phase

**Figure 4.**
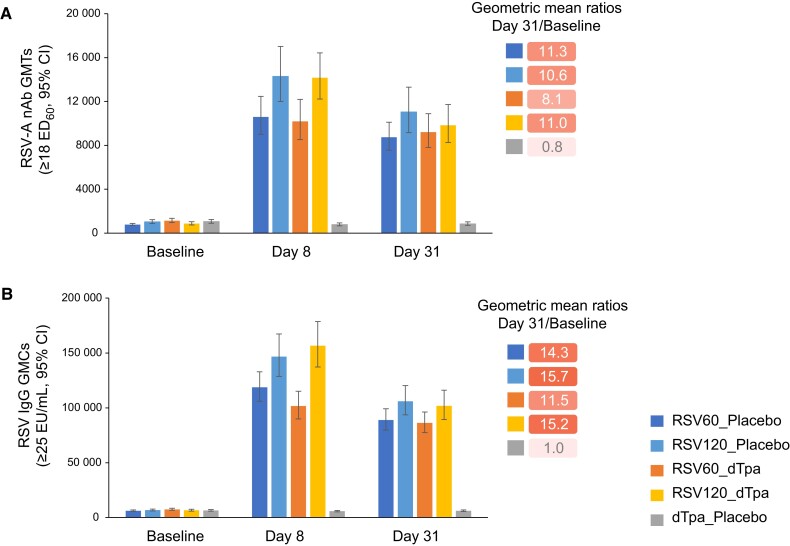
Humoral immune response in the primary phase to the 2 dose levels (60 and 120 μg) of RSVPreF3 in terms of (*A*) RSV-A neutralizing antibody geometric mean titers, and (*B*) RSV immunoglobulin G antibody geometric mean concentrations, at screening, day 8, and day 31 following first-dose vaccination, when given alone and coadministered with dTpa. Abbreviations: CI, confidence interval; dTpa, diphtheria, tetanus, and acellular pertussis; dTpa_placebo, participants who received dTpa and placebo; ED_60_, serum dilution inducing 60% inhibition in plaque-forming units; EU, enzyme-linked immunosorbent assay unit; GMC, geometric mean concentration; GMT, geometric mean titer; IgG, immunoglobulin G; nAb, neutralizing antibody; RSV, respiratory syncytial virus; RSV60_dTpa, participants who received RSV60 and dTpa; RSV60_placebo, participants who received RSV60 and placebo; RSV120_dTpa, participants who received RSV120 and dTpa; RSV120_placebo, participants who received RSV120 and placebo; RSVPreF3, RSV fusion protein stabilized in the prefusion conformation.

All participants had detectable anti-RSV-A nAbs and anti-RSVPreF3 IgG antibodies at baseline due to natural exposure to RSV. Anti-RSV-A nAb GMTs ranged from 771 (RSV60_placebo) to 1135 (RSV60_dTpa) (Figure 4*A*), and anti-RSVPreF3 IgG antibody GMCs ranged from 6172 (RSV60_placebo) to 7449 (RSV60_dTpa) (Figure 4*B*).

At day 8 and day 31, all RSVPreF3 groups showed a substantial increase in the immune response to RSVPreF3 (Figure 4 and Supplementary Table 7). Geometric mean ratios (GMRs; day 31/baseline) for RSV-A nAb and RSVPreF3 IgG antibody were close to 1.0 (0.7–1.0) in the dTpa_placebo group, and ranged from 8.1 to 15.7 across the 4 RSVPreF3 groups (Figure 4). Overall, analyses between groups showed no interference of dTpa coadministration on levels of anti-RSV-A nAb titers or anti-RSVPreF3 IgG antibody concentrations at day 31 after study vaccination.

##### Extension Phase

At 12–18 months after first vaccination, the immune response persisted in all groups and remained above prevaccination values. A second vaccination with RSVPreF3 120 μg induced a moderate increase in anti-RSV-A nAb titers (GMR range, 1.72–2.07) (Supplementary Table 8) and anti-RSVPreF3 IgG antibody concentrations (GMR range, 1.95–2.23), reaching somewhat lower levels compared to 1 month after first vaccination. Between-group analyses showed no difference in antibody responses between the RSVPreF3 primary dose levels, or when coadministered with either dTpa formulation.

#### Effect of RSVPreF3 Coadministration on Humoral Immune Responses to dTpa-Related Antigens

**Figure 5.**
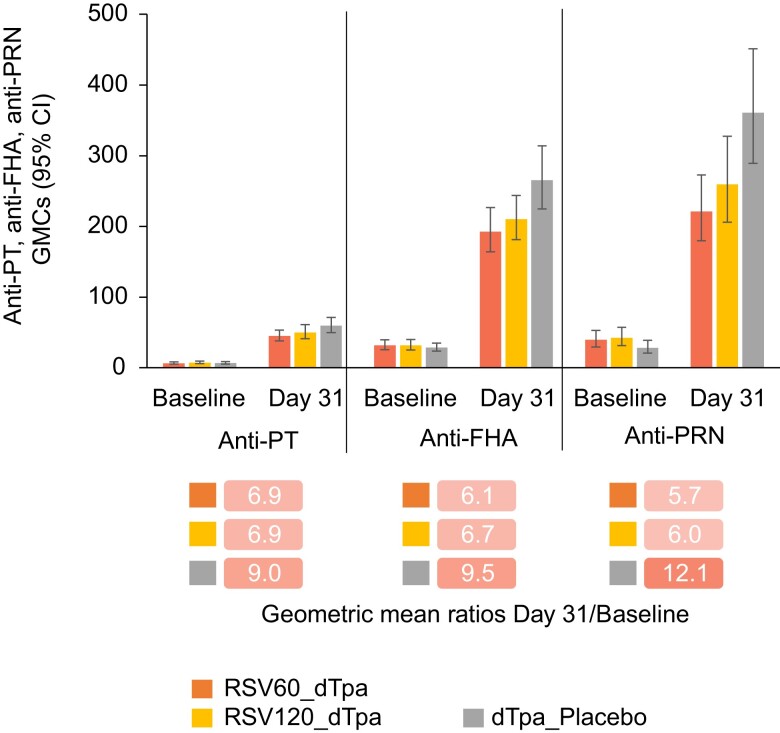
Geometric mean ratios (day 31/baseline) for titers of each pertussis antibody. Abbreviations: CI, confidence interval; dTpa, diphtheria, tetanus, and acellular pertussis; dTpa_placebo, participants who received dTpa and placebo; FHA, filamentous hemagglutinin; GMC, geometric mean concentration; PRN, pertactin; PT, pertussis toxoid; RSV, respiratory syncytial virus; RSV60_dTpa, participants who received RSV60 and dTpa; RSV120_dTpa, participants who received RSV120 and dTpa.

When coadministered with RSVPreF3, dTpa induced a lower immune response compared to dTpa alone. For each pertussis antigen, lower day 31 immune responses were observed in the 2 RSVPreF3 groups versus the dTpa_placebo group: GMCs of anti-PT antibodies (45.1–50.1 vs 59.5 IU/mL), anti-FHA antibodies (192.9–210.3 vs 265.6 IU/mL), and anti-PRN antibodies (221.3–259.6 vs 361.1 IU/mL) (Figure 5 and Supplementary Table 8).

GMCs of anti-D and anti-T antibodies were lower in the RSV120_dTpa and RSV60_dTpa groups than in the dTpa_placebo group at day 31 after vaccination (anti-D antibodies, 1.59–1.65 vs 2.42 IU/mL; anti-T antibodies, 5.76–5.83 vs 7.49 IU/mL). However, no group differences were observed between the RSV120_dTpa and dTpa_placebo groups, or between the RSV60_dTpa and dTpa_placebo groups, with respect to seroprotection rates provided by anti-D and anti-T antibodies (ie, the percentage of participants with anti-D or anti-T antibody concentrations ≥0.1 IU/mL by ELISA) at day 31 after vaccination, as 95% CIs for the group differences included zero (Supplementary Tables 6 and 9).

## DISCUSSION

The present data demonstrate that both RSVPreF3 dose levels (60 and 120 μg), regardless of whether administered with dTpa or placebo, had similar safety and reactogenicity profiles in healthy, nonpregnant women aged 18–45 years. A second vaccination with 120 μg RSVPreF3 administered 12–18 months after the first RSVPreF3 vaccination resulted in similar local and general safety profiles compared to the group receiving RSVPreF3 120 μg for the first time (dTpa_placebo_RSV120). Injection-site pain was reported at a higher frequency following the second vaccination, a common finding with other vaccines [14]; however, the gap between first and second doses in this study was longer than generally seen with other multidose vaccines. Similar safety profiles were observed in the study groups that received RSVPreF3 120 μg as a first or second vaccination. No safety concerns were identified in this study that would preclude further development of RSVPreF3, administered as a single intramuscular dose (either 60 or 120 μg) with dTpa, or administered as a second dose (120 μg), in women aged 18–45 years. The overall safety profile of dTpa was in line with that reported in previously published studies [15, 16].

RSVPreF3 coadministered with dTpa induced a robust immune response, demonstrating no interference of dTpa with response to RSVPreF3. Both doses of RSVPreF3, when administered with dTpa or placebo, induced substantial increases in RSV-A nAb titers and anti-RSVPreF3 IgG antibody concentrations at 1 month postvaccination, persisting for up to 12–18 months. A second vaccination with 120 μg RSVPreF3 induced moderate increases in RSV-A nAb titers and anti-RSVPreF3 IgG antibody concentrations compared to levels before the second vaccination (GMRs of around 2). While antibody levels after the second vaccination were lower compared with first vaccination, they remained >5 times above baseline. Lower booster responses have also been observed with other RSV vaccines [17]. It is possible that the presence of preexisting neutralizing antibodies partially interfere with the immune response to second vaccination, a phenomenon observed with mRNA vaccines [18].

Interference of RSVPreF3 on the immune response to the components of dTpa was observed at 1 month postvaccination. For the diphtheria and tetanus antigens, the lower antibody responses are not considered to have clinical relevance in view of the similar seroprotection rates between the study groups and similar seroprotection rates to a primary infant dTpa vaccination series [19]. The clinical significance of the lower antibody response to pertussis antigens in the study groups coadministered with either dose level of RSVPreF3 remains unclear, as no seroprotective threshold is currently established for pertussis.

Another F protein vaccine (Abrysvo) for RSV has also been investigated with coadministration and has reported similar findings to our study [20–22]. Coadministration of Abrysvo with tetanus toxoid, reduced diphtheria toxoid, and acellular pertussis vaccine (Tdap) resulted in reduced humoral immune responses to pertussis antigens compared with administration of Tdap alone, failing to achieve noninferiority criteria [21]. Coadministration with the seasonal influenza vaccine did not affect immunogenicity of Abrysvo, but humoral immune responses to the influenza vaccine were reduced compared with administration of the vaccines alone [20, 22]. The clinical significance and mechanisms of these reductions are unknown and further study is required [20, 21].

Study limitations include that participants were females, aged ≤45 years and most were white, which may limit generalizability of the results to the broader population; in the extension phase, the number of participants receiving RSVPreF3 120 μg as a second vaccination was small, thus limiting the potential impact of between-group comparisons. Nevertheless, the primary and extension phases provide valuable early data about the potential future clinical utility of an investigational maternal RSV vaccine to protect neonates against RSV-associated acute LRTI.

While results from this study support further development of the vaccine, enrollment and vaccination in all studies evaluating the RSVPreF3 maternal vaccine were stopped because of an imbalance in preterm births and associated neonatal deaths observed in the phase 3 study RSV MAT-009 (GRACE). GSK has discontinued any additional work on the maternal RSVPreF3 and no new participants will be vaccinated in this program.

## CONCLUSIONS

In summary, these results reveal no safety concerns for coadministration of a single intramuscular dose of RSVPreF3 60 or 120 µg with dTpa (irrespective of dTpa formulation) or placebo, or for a second vaccination with RSVPreF3 120 µg, in healthy, nonpregnant women aged 18–45 years. Both RSVPreF3 dose levels (60 and 120 µg) considerably increased RSV-A nAb titers and anti-RSVPreF3 IgG antibody concentrations at 1 month postvaccination, and these increases persisted up to 12–18 months postvaccination. Coadministration of dTpa (irrespective of formulation) did not interfere with the immune response to RSVPreF3. Conversely, while RSVPreF3 interfered with the immune response to dTpa, particularly regarding the response to pertussis antigens, the latter response remained robust and is unlikely to be clinically relevant. Overall, the safety, reactogenicity, and immunogenicity data presented here support a favorable benefit-risk profile for maternal vaccination with either RSVPreF3 dose level.

## Supplementary Data

Supplementary materials are available at *The Journal of Infectious Diseases* online (http://jid.oxfordjournals.org/). Supplementary materials consist of data provided by the author that are published to benefit the reader. The posted materials are not copyedited. The contents of all supplementary data are the sole responsibility of the authors. Questions or messages regarding errors should be addressed to the author.

## Supplementary Material

jiad560_Supplementary_Data

## Notes

***Acknowledgments*.** Nathalie Baudson contributed to all clinical laboratory management aspects in the study. We thank all study participants as well as the staff at the participating clinical sites. Medical writing support was provided by Lewis Cawkwell from Apollo, OPEN Health Communications, funded by GSK.

***Author contributions.*** J. H. K. and I. D. contributed to the conceptualization of the study. N. He., I. L.-R., M. F., and S. P. contributed to data collection or generation. P. v. d. S., B. A., N. Hu., and D. Y. contributed to methodology. N. He., N. Hu., and D. Y. contributed to formal analyses. N. He., I. L.-R., N. Hu., and I. D. were involved in data analysis and interpretation. All authors contributed to manuscript writing, review, and editing, and approved the final version of the manuscript.

***Data availability*.** Anonymized individual participant data and study documents can be requested for further research from https://www.clinicalstudydatarequest.com/Study-Sponsors/Study-Sponsors-GSK.aspx.

***Financial support*.** This work was supported by GlaxoSmithKline.
