# Supplementary material for: Safety and Immunogenicity of Respiratory Syncytial Virus Prefusion Maternal Vaccine Coadministered With Diphtheria-Tetanus-Pertussis Vaccine: A Phase 2 Study
Source: J Infect Dis. 2023 Dec 22;230(2):e353–62. doi: 10.1093/infdis/jiad560 (PMC11326842; doi:10.1093/infdis/jiad560)
Supplement: jiad560_Supplementary_Data [file jiad560_supplementary_data.zip › RSV_MAT011_final draft_14Nov23_Supplement resub Clean.docx]

**SUPPLEMENTARY MATERIALS**

**METHODS**

**Vaccine Composition, Dosage, and Route of Administration**

RSVPreF3 (GSK388550A) 60 or 120 μg (0.5 mL volume) was given intramuscularly with placebo (150 mM sodium chloride), or co-administered with a single intramuscular dose of combined reduced-antigen-content dTpa vaccine (*Boostrix*; US formulation SB776423, or ex-US formulation SB263855). Two dTpa formulations were administered because of differences in licensing requirements within and outside the US (US: dTpa_300; ex-US: dTpa_500). All RSVPreF3 first vaccinations were administered to the left arm, whereas placebo and dTpa were administered to the right arm, except in participants receiving dTpa_Placebo, where placebo was administered in the left arm and dTPa in the right. In the extension phase, the second dose of RSVPreF3 120 μg was administered in the non-dominant arm.

**Randomization**

A total of approximately 500 eligible participants (250 in the US, and 250 ex-US) were planned to be enrolled. Of these, approximately 250 participants were planned to be randomized to five US study groups in a 1:1:1:1:1 ratio using source database for internet randomization (SBIR), and the remaining approximately 250 participants were planned to be randomized to five ex-US study groups in a 1:1:1:1:1 ratio using SBIR. The randomization algorithm used a minimization procedure by treating study formulation (US or ex-US) as a stratification factor; and age at the time of vaccination (18–32 or 33–45 years), and center, as minimization factors.

**Other Eligibility Criteria**

For both the primary and extension phases, women of childbearing potential were eligible if they had: practiced adequate contraception for 30 days prior to the first and second vaccinations; a negative pregnancy test on the days of the first and second vaccinations; agreed to continue adequate contraception for 90 days after completion of the vaccination series.

Exclusion criteria included:

- A history of medical conditions such as: any reaction or hypersensitivity likely to be exacerbated by any component of the vaccines; any immunosuppressive or immunodeficient condition; hypersensitivity to latex; major congenital defects; pulmonary, cardiovascular, hepatic, or renal functional abnormality; significant, or uncontrolled, psychiatric illness; recurrent or uncontrolled neurological disorders/seizures; human immunodeficiency virus-positive status; autoimmune disease; body mass index (BMI) >40 kg/m^2^; any clinically significant hematological parameter and/or biochemical laboratory abnormality.
- Prior use of: any investigational or non-registered product (other than the study vaccines), or any long-acting immune-modifying drugs during the study period; administration of immunoglobulins/blood products/plasma derivatives 3 months before the first dose of study vaccines or planned administration during study period; ≥14 days in total of immunosuppressants or other immune-modifying drugs 3 months prior to the first vaccine dose (for corticosteroids, this will mean prednisone ≥5 mg/day, or equivalent; inhaled and topical steroids are allowed); planned administration/administration of a vaccine not foreseen by the study protocol within the period starting 30 days before and ending 30 days after study primary vaccination, with the exception of any licensed influenza vaccine which may be administered ≥15 days before or after study vaccination; administration of a vaccine containing diphtheria, tetanus or pertussis antigens or diphtheria and tetanus toxoids within the previous 5 years; previous experimental vaccination against RSV.

**Additional Primary Immunogenicity Endpoint Analyses Methods**

Antibody titer/concentrations were displayed using reverse cumulative curves pooled. The kinetics of GMT/GMCs were plotted as a function of time for subjects with results available at all time points pooled. GMT/GMC ratios and their 90% CI between RSVPreF3+dTpa and RSVPreF3 alone in terms of RSV-A nAb titers and RSV IgG antibody concentrations were calculated at Screening, Day 8, and Day 31, and pooled for all participants.

*Assessment Powering*

The sample size of 50 subjects per group and 100 subjects per group in the combined formulations at the first vaccination were planned to provide reasonable confidence to evaluate the adverse event (AE) rate and quantify immunogenicity interference. If an AE is not observed in a given treatment group, a sample size of 50 subjects can provide at least 95% confidence to rule out an AE incidence greater than 7.1%, and a total of 100 subjects in the combined formulations can provide at least 95% confidence to rule out an AE rate greater than 3.6%. A combined 400 subjects across the RSVPreF3 groups and formulations can provide at least 95% confidence to rule out an AE incidence rate greater than 0.9%. Furthermore, a sample size of 50 subjects per group and 100 subjects per group in the combined formulations would provide a probability of 92% or 99% to observe at least one AE respectively, if the true AE rate is 5%. In addition, a sample size of 400 when combining the RSVPreF3 groups would provide a probability of 98% to observe at least one AE if the true AE rate is only 1%. No hypothesis driven sample size calculation was conducted due to the descriptive nature of the study.

For the humoral immune response, if a standard deviation of log10 transformed RSV-A Nab of 0.4 is assumed, when the sample size is 50 subjects per group, the half width of the 90% CI on the log ratio of GMT between co-administration with dTpa and RSVPreF3 alone is 0.133. With an observed GMT ratio of 1, its 90% CI will be (0.74, 1.36). With subjects in US and ex-US combined, there will be at least 90% chance that the upper bound of one-sided 95% CI for the ratio of GMT of RSV-A Nab titer between RSVPreF3 alone and co-administration with dTpa is under 1.5-fold.

**Secondary Endpoint Analyses Methods**

*Safety Analyses*

For each group with first-dose vaccination of RSVPreF3 (two-dose level) when given alone or co-administered with dTpa, the following points were analyzed for the first- and second-dose vaccination.

- The percentage of participants with at least one local AE and with at least one general AE and with any (“solicited or unsolicited”) AE during the 7-day or 30-day follow-up, as well as any SAE up to a period of 180 days post-first and -second vaccination time points, were tabulated with exact 95% CI by study group. The same computations were done for Grade 3 AEs, for any AEs considered related to vaccination, for Grade 3 AEs considered related to vaccination, and for AEs resulting in medically attended visits.
- The percentage of subjects with at least one report of SAE classified by the MedDRA System Organ Class (SOC) and Preferred Terms, and reported during the 30-day follow-up period, were tabulated with exact 95% CI for each formulation (US and ex-US).
- The percentage of subjects reporting each individual solicited local AE (any grade, Grade 3, and those resulting in a medically attended visit) and solicited general AE (any grade, Grade 3, related, Grade 3 related, and those resulting in a medically attended visit) during the 7-day follow-up period (ie, on the day of vaccination and six subsequent days) were tabulated based on maximum intensity for each group for each formulation (US and ex-US).
- For fever, the number and percentage of subjects reporting fever by half-degree (°C) cumulative increments during the 7-day follow-up period were tabulated for each group for each formulation (US and ex-US). Similar tabulations were performed for any fever with a causal relationship to vaccination, Grade 3 or above (>39.0°C/102.2°F) causally related fever, and fever resulting in a medically attended visit. In addition, the prevalence of any and Grade 3 fever were presented graphically over time after each vaccination.
- For each formulation (US and ex-US), the percentage of subjects with any unsolicited AEs during the 30-day follow-up period (ie, on the day of vaccination and 29 subsequent days) with its exact 95% CI were tabulated by group and by MedDRA SOC and Preferred Term. Similar tabulations were done for Grade 3 unsolicited AEs, for any causally related unsolicited AEs, for Grade 3 causally related unsolicited AEs, and for unsolicited AEs resulting in a medically attended visit. The verbatim reports of unsolicited AEs were reviewed by a physician, and the signs and symptoms were coded according to the MedDRA Dictionary for Adverse Reaction Terminology.

*Immunogenicity Analyses*

For each group, at each time point that blood samples are collected for humoral immune response and for each assay (unless otherwise specified):

- GMCs/GMTs and their 95% CI were tabulated and represented graphically by formulation (US and ex-US). Geometric mean of ratios of antibody titer/concentrations at each individual post-vaccination time point over pre-vaccination (screening) were tabulated with 95% CI by formulation (US and ex-US).
- Antibody titer/concentration were displayed using reverse cumulative curves by formulation (US and ex-US).
- The kinetics of GMT/GMCs were plotted as a function of time for subjects with results available at all time points by formulation (US and ex-US).
- Booster response rates for PT, FHA, and PRN (with exact 95% CI) were calculated by group for each formulation (US and ex-US).

For the data collected for the second vaccination, the humoral immune response of RSVPreF3 was summarized based on two dose levels (60 and 120 μg) from the first vaccination.

- GMCs/GMTs and their 95% CI were tabulated and represented graphically for prior and post-second dose vaccination.
- GMRs of antibody titer/concentrations at individual post-second dose vaccination time points (Visit 6) over prior second dose vaccination time points (Visit 4) were tabulated with 95% CI.
- Antibody titer/concentration were displayed using reverse cumulative curves on prior and post-second vaccination.
- The kinetics of GMT/GMCs were plotted as a function of time for subjects with results available at all time points including the visits from the first vaccination.

Booster responses to PT, FHA, and PRN antigens are defined as:

- For subjects with pre-vaccination antibody concentration below the assay cut-offs: post-vaccination antibody concentration ≥4 times the assay cut-offs,
- For subjects with pre-vaccination antibody concentration between the assay cut-offs and below four times the assay cut-offs: post-vaccination antibody concentration ≥4 times the pre-vaccination antibody concentration, and
- For subjects with pre-vaccination antibody concentration ≥4 times the assay cut-offs: post-vaccination antibody concentration ≥2 times the pre-vaccination antibody concentration:
  - The percentage of subjects with anti-D antibody concentrations ≥1.0 IU/mL by ELISA and the percentage of subjects with anti-T antibody concentrations ≥1.0 IU/mL by ELISA (with exact 95% CI) were calculated by group for each formulation (US and ex-US).
  - The GMT/GMCs ratio and their 90% CI between RSVPreF3+dTpa and dTpa_Placebo in terms of anti-PT, anti-FHA, anti-PRN, anti-D, and anti-T were calculated at Screening and Day 31 pooled for all subjects and by formulation (US and ex-US).
  - A further exploratory between-groups analysis was performed at Day 31 using an Analysis of Covariance (ANCOVA) model by including study groups, study formulation (US and ex-US), age category, and level of antibodies at Screening as covariates.
  - For *Boostrix* booster response to antigens PT, FHA, and PRN, the two-sided standardized asymptotic 95% CI for the group differences in the percentage of subjects with a booster response to each antigen in the RSVPreF3+dTpa vaccine groups and (minus) the dTpa_Placebo vaccine group on Day 31 post-vaccination was calculated for each formulation.

For *Boostrix* seroprotection rate (percentage of subjects with antibody concentrations ≥0.1 IU/mL by ELISA) for antigens D and T, the two-sided standardized asymptotic 95% CI for the group differences in the percentage of subjects with antibody concentrations ≥0.1 IU/mL in the RSVPreF3+dTpa vaccine groups and (minus) the dTpa_Placebo vaccine group at Day 31 post-vaccination was calculated for each formulation.

**RESULTS**

**Extension Phase Safety Results**

The reported Grade 3 unsolicited AEs were pyelonephritis, tonsillitis, abdominal pain, diarrhea, vomiting, pain, and muscle strain, each reported by one participant (0.5%).

The reported medically attended AEs were bronchitis, nasopharyngitis, pharyngitis, pyelonephritis, tonsillitis, upper respiratory tract infection, ear pain, abdominal pain, diarrhea, vomiting, contusion, rash, and hypertension, each reported by one participant (0.5%).

Unsolicited Adverse Events

Reports of unsolicited AEs were evenly distributed across the study groups, ranging from 18% (RSV120_dTpa_RSV120) to 29% (RSV60_Placebo_RSV120). The most frequently reported unsolicited AEs were headache (4%; n=8), fatigue (2%; n=4), and oropharyngeal pain (2%; n=4) (**Supplementary Table 5**). The most common vaccine-related unsolicited AEs were injection-site pruritus by three participants (1%), injection-site pain, malaise, and myalgia, each reported by two participants (1%).

Five participants (2%) across the study groups reported Grade 3 unsolicited AEs (Supplementary Materials). One case of Grade 3-related pain in the RSV60_Placebo_RSV120 group was the only vaccine-related unsolicited AE. Ten participants (5%) across the study groups reported medically attended AEs (Supplementary Materials).

*Serious Adverse Events*

Four SAEs were reported by two participants, none were vaccine-related: one participant in the RSV120_dTpa group reported three SAEs (radius, ulna, and wrist fracture) 88 days after the first vaccination; and one participant in the RSV60_Placebo_RSV120 group had a serious case of pyelonephritis 1 day after the second vaccination. There were no fatalities, and no withdrawals from the study due to AEs.

*Pregnancies*

All four pregnancies (RSV60_Placebo_RSV120; dTpa_Placebo_RSV120; RSV120_Placebo; RSV60_dTpa n=1 each) reported at the time of the final database freeze (April 5, 2022) resulted in live births at term with no apparent congenital anomalies.

**Supplementary Figures**

**Supplementary Figure 1.** Consolidated Standards of Reporting Trials (CONSORT) flow chart of participant enrollment, group allocation, and elimination/exclusions in the extension phase.

Abbreviations: dTpa, diphtheria, tetanus, and acellular pertussis; dTpa_Placebo_RSV120, participants who received dTpa and placebo in the primary phase; ICF, informed consent form; PPS, Per Protocol Set; RSV, respiratory syncytial virus; RSV60_dTpa_RSV120, participants who received RSV60 and dTpa in the primary phase; RSV60_Placebo_RSV120, participants who received RSV60 and placebo in the primary phase; RSV120_dTpa_RSV120, participants who received RSV120 and dTpa in the primary phase; RSV120_Placebo_RSV120, participants who received RSV120 and placebo in the primary phase.

**Supplementary Figure 2.** Most frequent unsolicited AEs reported during Days 1–30 following the first vaccination, pooled for dTpa formulation.

Abbreviations: AE, adverse event; dTpa, diphtheria, tetanus, and acellular pertussis; dTpa_Placebo, participants who received dTpa and placebo; RSV, respiratory syncytial virus; RSV60_dTpa, participants who received RSV60 and dTpa; RSV60_Placebo, participants who received RSV60 and placebo; RSV120_dTpa, participants who received RSV120 and dTpa; RSV120_Placebo, participants who received RSV120 and placebo; RTI, respiratory tract infection.
