# Supplementary material for: Safety and Immunogenicity of Respiratory Syncytial Virus Prefusion Maternal Vaccine Coadministered With Diphtheria-Tetanus-Pertussis Vaccine: A Phase 2 Study
Source: J Infect Dis. 2023 Dec 22;230(2):e353–62. doi: 10.1093/infdis/jiad560 (PMC11326842; doi:10.1093/infdis/jiad560)
Supplement: jiad560_Supplementary_Data [file jiad560_supplementary_data.zip › Supplementary_Table_1.docx]

**Supplementary Table 1.** Study Objectives and Endpoints

| **Objectives** | **Endpoints** |
| --- | --- |
| **Primary** | |
| ***Safety*** |  |
| To evaluate the safety and reactogenicity of two dose levels (60 and 120 μg) of RSVPreF3 when given alone or co-administered with dTpa from vaccination up to Day 31. | Occurrence of any AEs from vaccination to Day 31:   - Occurrence of each solicited local AE at the site of injection in both limbs from vaccination to Day 8 - Occurrence of solicited general AEs from vaccination to Day 8 - Occurrence of any unsolicited AEs from vaccination to Day 31 - Occurrence of SAEs from vaccination to Day 31. |
| To evaluate the safety of a second dose of RSVPreF3 given from 12 up to 18 months post-first dose up to Day 31 days post-second dose vaccination. | Occurrence of any AEs from second dose to Day 31 post-second dose vaccination, for all subjects:   - Occurrence of each solicited local AE at the site of injection in both deltoids from second dose vaccination to Day 8 post-second dose vaccination - Occurrence of solicited general AEs from second dose vaccination to Day 8 post-second dose vaccination - Occurrence of any unsolicited AEs from second dose to Day 31 post-second dose vaccination - Occurrence of SAEs from second dose vaccination to Day 31 post-second dose vaccination. |
| ***Immunogenicity*** |  |
| To evaluate the humoral immune response to two dose levels (60 and 120 μg) of RSVPreF3 when given alone and co-administered with dTpa, at Screening, Day 8, and Day 31 post-first dose vaccination. | RSV-A neutralizing antibody titers at Screening, Day 8, and Day 31 in all groups   - RSV IgG antibody concentrations at Screening, Day 8, and Day 31. |
| **Secondary** | |
| ***Safety*** | |
| To evaluate the safety and reactogenicity of two dose levels (60 and 120 μg) of RSVPreF3 when given alone or co-administered with dTpa from first dose vaccination up to Day 31 by formulation. | Occurrence of any AEs from vaccination to Day 31, for all subjects:   - Occurrence of each solicited local AE at the site of injection in both limbs from vaccination to Day 8 - Occurrence of solicited general AEs from vaccination to Day 8 - Occurrence of any unsolicited AEs from vaccination to Day 31 - Occurrence of SAEs from vaccination to Day 31. |
| To evaluate the safety of two dose levels (60 and 120 μg) of RSVPreF3 when given alone and co-administered with dTpa compared to dTpa_Placebo groups from first dose vaccination up to Day 181. | Occurrence of SAEs from vaccination up to Day 181. |
| To evaluate the safety of two dose levels (60 and 120 μg) of RSVPreF3 when given alone and co-administered from vaccination up to Day 181 by formulation. | Occurrence of SAEs from vaccination up to Day 181. |
| To evaluate the safety of RSVPreF3 (pooled for all groups receiving RSVPreF3) for the first dose vaccination. | Occurrence of SAEs from vaccination up to Day 181. |
| To evaluate the safety of second dose of RSVPreF3 given from 12 to up to 18 months post-first dose vaccination up to Day 181 post-second dose vaccination. | Occurrence of SAEs from second dose vaccination up to Day 181 post-second dose vaccination. |
| ***Immunogenicity*** | |
| To evaluate the humoral immune response and persistence to two dose levels (60 and 120 μg) of RSVPreF3 when given alone and co-administered with dTpa at Screening, Day 8, and Day 31 post-first dose vaccination, and at 12 to 18 months by formulation. | RSV-A neutralizing antibody titers at Screening, Day 8, Day 31, and a single timepoint between 12 to 18 months post-first vaccination.  RSV IgG antibody concentrations at Screening, Day 8, Day 31, and a single timepoint between 12 to 18 months post-first vaccination. |
| To evaluate the humoral immune response to the pertussis component of the dTpa vaccine when given alone and co-administered with two dose levels (60 and 120 μg) of RSVPreF3 at Screening and Day 31 post-first dose vaccination by formulation. | Antibody concentrations against pertussis toxoid (anti-PT), filamentous hemagglutinin (anti-FHA), and pertactin (anti-PRN) concentrations at Screening and Day 31. |
| To evaluate the humoral immune response to the diphtheria (D) component of the dTpa vaccine when given alone and co-administered with two dose levels (60 and 120 μg) of RSVPreF3 at Screening and Day 31 post-first dose vaccination by formulation. | Anti-D concentrations at Screening and Day 31. |
| To evaluate the humoral immune response to the tetanus (T) component of the dTpa vaccine when given alone and co-administered with two dose levels (60 and 120 μg) of RSVPreF3 at Screening and Day 31 post-first dose vaccination by formulation. | Anti-T concentrations at Screening and Day 31. |
| To evaluate the humoral immune response of a second dose vaccination of RSVPreF3 (120 μg), following a first dose vaccination of either 60 or 120 μg. | RSV-A neutralizing antibody titers concentrations 31 days post-second dose vaccination.  • RSV IgG antibody concentrations 31 days post-second dose vaccination. |

Abbreviations: AE, adverse event; dTPA, diphtheria, tetanus, and acellular pertussis; dTpa_Placebo, participants who received dTpa and placebo; IgG, immunoglobulin G; RSV, respiratory syncytial virus; RSVPreF3, RSV fusion protein stabilized in the prefusion conformation; SAE, serious adverse event.
