# Supplementary material for: Safety and Immunogenicity of Respiratory Syncytial Virus Prefusion Maternal Vaccine Coadministered With Diphtheria-Tetanus-Pertussis Vaccine: A Phase 2 Study
Source: J Infect Dis. 2023 Dec 22;230(2):e353–62. doi: 10.1093/infdis/jiad560 (PMC11326842; doi:10.1093/infdis/jiad560)
Supplement: jiad560_Supplementary_Data [file jiad560_supplementary_data.zip › Supplementary_Table_2.docx]

**Supplementary Table 2.** Summary of Participant Characteristics Pooled for dTpa formulation (Extension Phase) — Exposed Set

|  | RSV120_dTpa_RSV120  (n=39) | RSV120_Placebo_RSV120  (n=41) | RSV60_dTpa_RSV120  (n=46) | RSV60_Placebo_RSV120  (n=41) | dTpa_Placebo_RSV120  (n=46) | Total  (N=213) |
| --- | --- | --- | --- | --- | --- | --- |
| Age at vaccination, mean (SD), years | 32.1 (7.9) | 31.7 (8.4) | 30.4 (8.6) | 30.3 (8.4) | 32.6 (8.2) | 31.4 (8.3) |
| Age category at vaccination, n (%) |  |  |  |  |  |  |
| 18–32 years | 21 (53.8) | 23 (56.1) | 25 (54.3) | 25 (61.0) | 19 (41.3) | 113 (53.1) |
| 33–45 years | 18 (46.2) | 18 (43.9) | 21 (45.7) | 16 (39.0) | 27 (58.7) | 100 (46.9) |
| Country, n (%) |  |  |  |  |  |  |
| Belgium | 12 (30.8) | 15 (36.6) | 17 (37.0) | 16 (39.0) | 16 (34.8) | 76 (35.7) |
| Canada | 13 (33.3) | 16 (39.0) | 15 (32.6) | 13 (31.7) | 15 (32.6) | 72 (33.8) |
| United States | 14 (35.9) | 10 (24.4) | 14 (30.4) | 12 (29.3) | 15 (32.6) | 65 (30.5) |

| Ethnicity, n (%) |  |  |  |  |  |  |
| --- | --- | --- | --- | --- | --- | --- |
| Hispanic or Latinx | 3 (7.7) | 0 (0.0) | 0 (0.0) | 2 (4.9) | 3 (6.5) | 8 (3.8) |
| Not Hispanic or Latinx | 36 (92.3) | 41 (100.0) | 46 (100.0) | 39 (95.1) | 43 (93.5) | 205 (96.2) |
| Race, n (%) |  |  |  |  |  |  |
| American Indian or Alaska Native | 1 (2.6) | 0 (0.0) | 0 (0.0) | 0 (0.0) | 0 (0.0) | 1 (0.5) |
| Asian | 1 (2.6) | 0 (0.0) | 1 (2.2) | 2 (4.9) | 0 (0.0) | 4 (1.9) |
| Black or African American | 0 (0.0) | 0 (0.0) | 1 (2.2) | 0 (0.0) | 1 (2.2) | 2 (0.9) |
| Native Hawaiian or other Pacific Islander | 0 (0.0) | 0 (0.0) | 0 (0.0) | 0 (0.0) | 0 (0.0) | 0 (0.0) |
| White | 36 (92.3) | 41 (100.0) | 42 (91.3) | 39 (95.1) | 44 (95.7) | 202 (94.8) |
| Other | 1 (2.6) | 0 (0.0) | 2 (4.3) | 0 (0.0) | 1 (2.2) | 4 (1.9) |

Abbreviations: dTPA, diphtheria, tetanus, and acellular pertussis; dTpa_Placebo_RSV120, participants who received dTpa and placebo in the primary phase; N, number of participants; RSV, respiratory syncytial virus; RSV60_dTpa_RSV120, participants who received RSV60 and dTpa in the primary phase; RSV60_Placebo_RSV120, participants who received RSV60 and placebo in the primary phase; RSV120_dTpa_RSV120, participants who received RSV120 and dTpa in the primary phase; RSV120_Placebo_RSV120, participants who received RSV120 and placebo in the primary phase; SD, standard deviation.
