# Supplementary material for: Safety and Immunogenicity of Respiratory Syncytial Virus Prefusion Maternal Vaccine Coadministered With Diphtheria-Tetanus-Pertussis Vaccine: A Phase 2 Study
Source: J Infect Dis. 2023 Dec 22;230(2):e353–62. doi: 10.1093/infdis/jiad560 (PMC11326842; doi:10.1093/infdis/jiad560)
Supplement: jiad560_Supplementary_Data [file jiad560_supplementary_data.zip › Supplementary_Table_3.docx]

**Supplementary Table 3.** Percentage of Subjects with Solicited Local AEs Associated With RSV and/or Placebo on Left Arm, and General AEs Within 7 days of the First Vaccination (Primary Phase) — Solicited Safety Set

|  | RSV120_dTpa  (n=101) | RSV120_Placebo  (n=101) | RSV60_dTpa  (n=103) | RSV60_Placebo  (n=102) | dTpa_Placebo  (n=99) |
| --- | --- | --- | --- | --- | --- |
| **Local AEs** |  |  |  |  |  |
| Any erythema, n (%) | 6 (5.9) | 5 (5.0) | 6 (5.8) | 6 (5.9) | 0 |
| 95% CI | (2.2, 12.5) | (1.6, 11.3) | (2.2, 12.2) | (2.2, 12.4) | (0, 3.7) |
| Any pain, n (%) | 52 (51.5) | 54 (54.0) | 54 (52.4) | 60 (58.8) | 19 (19.2) |
| 95% CI | (41.3, 61.6) | (43.7, 64.0) | (42.4, 62.4) | (48.6, 68.5) | (12.0, 28.3) |
| Any swelling, n (%) | 2 (2.0) | 7 (7.0) | 3 (2.9) | 5 (4.9) | 0 |
| 95% CI | (0.2, 7.0) | (2.9, 13.9) | (0.6, 8.3) | (1.6, 11.1) | (0, 3.7) |
| **General AEs** |  |  |  |  |  |
| Any fatigue, n (%) | 41 (40.6) | 40 (39.6) | 39 (37.9) | 33 (32.4) | 38 (38.4) |
| 95% CI | (30.9, 50.8) | (30.0, 49.8) | (28.5, 48.0) | (23.4, 42.3) | (28.8, 48.7) |
| Any GI symptoms, n (%) | 24 (23.8) | 29 (28.7) | 28 (27.2) | 29 (28.4) | 28 (28.3) |
| 95% CI | (15.9, 33.3) | (20.1, 38.6) | (18.9, 36.8) | (19.9, 38.2) | (19.7, 38.2) |
| Any headache, n (%) | 45 (44.6) | 46 (45.5) | 36 (35.0) | 40 (39.2) | 37 (37.4) |
| 95% CI | (34.7, 54.8) | (35.6, 55.8) | (25.8, 45.0) | (29.7, 49.4) | (27.9, 47.7) |
| Any temperature, n (%) | 2 (2.0) | 4 (4.0) | 3 (2.9) | 4 (3.9) | 7 (7.1) |
| 95% CI | (0.2, 7.0) | (1.1, 9.8) | (0.6, 8.3) | (1.1, 9.7) | (2.9, 14.0) |

Abbreviations: AE, adverse event; CI, confidence interval; dTPA, diphtheria, tetanus, and acellular pertussis; dTpa_Placebo, participants who received dTpa and placebo; GI, gastrointestinal; N, number of participants; RSV, respiratory syncytial virus; RSV60_dTpa, participants who received RSV60 and dTpa; RSV60_Placebo, participants who received RSV60 and placebo; RSV120_dTpa, participants who received RSV120 and dTpa; RSV120_Placebo, participants who received RSV120 and placebo.

Note: All solicited local (injection-site) reactions were considered causally related to vaccination, as per protocol.
