# Supplementary material for: Safety and Immunogenicity of Respiratory Syncytial Virus Prefusion Maternal Vaccine Coadministered With Diphtheria-Tetanus-Pertussis Vaccine: A Phase 2 Study
Source: J Infect Dis. 2023 Dec 22;230(2):e353–62. doi: 10.1093/infdis/jiad560 (PMC11326842; doi:10.1093/infdis/jiad560)
Supplement: jiad560_Supplementary_Data [file jiad560_supplementary_data.zip › Supplementary_Table_4.docx]

**Supplementary Table 4.** Percentage of Subjects with Solicited Local AEs Associated with RSV, and General AEs Within 7 Days of the Second Vaccination (Extension Phase) — Solicited Safety Set

|  | RSV120_dTpa_RSV120  (n=39) | RSV120_Placebo_RSV120  (n=39) | RSV60_dTpa_RSV120  (n=46) | RSV60_Placebo_RSV120  (n=41) | dTpa_Placebo_RSV120  (n=44) |
| --- | --- | --- | --- | --- | --- |
| **Local AEs** |  |  |  |  |  |
| Any erythema, n (%) | 7 (17.9) | 4 (10.3) | 4 (8.7) | 6 (14.6) | 1 (2.3) |
| 95% CI | (7.5, 33.5) | (2.9, 24.2) | (2.4, 20.8) | (5.6, 29.2) | (0.1, 12.0) |
| Any pain, n (%) | 34 (87.2) | 34 (87.2) | 37 (80.4) | 35 (85.4) | 17 (38.6) |
| 95% CI | (72.6, 95.7) | (72.6, 95.7) | (66.1, 90.6) | (70.8, 94.4) | (24.4, 54.5) |
| Any swelling, n (%) | 5 (12.8) | 3 (7.7) | 4 (8.7) | 2 (4.9) | 1 (2.3) |
| 95% CI | (4.3, 27.4) | (1.6, 20.9) | (2.4, 20.8) | (0.6, 16.5) | (0.1, 12.0) |
| **General AEs** |  |  |  |  |  |
| Any fatigue, n (%) | 10 (25.6) | 13 (33.3) | 17 (37.0) | 19 (46.3) | 15 (34.1) |
| 95% CI | (13.0, 42.1) | (19.1, 50.2) | (23.2, 52.5) | (30.7, 62.6) | (20.5, 49.9) |
| Any GI symptoms, n (%) | 4 (10.3) | 8 (20.5) | 7 (15.2) | 10 (24.4) | 6 (13.6) |
| 95% CI | (2.9, 24.2) | (9.3, 36.5) | 6.3, 28.9) | (12.4, 40.3) | (5.2, 27.4) |
| Any headache, n (%) | 11 (28.2) | 13 (33.3) | 15 (32.6) | 23 (56.1) | 14 (31.8) |
| 95% CI | (15.0, 44.9) | (19.1, 50.2) | (19.5, 48.0) | (39.7, 71.5) | (18.6, 47.6) |
| Any temperature, n (%) | 0 | 0 | 1 (2.2) | 4 (9.8) | 1 (2.3) |
| 95% CI | (0, 9.0) | (0, 9.0) | (0.1, 11.5) | (2.7, 23.1) | (0.1, 12.0) |

Abbreviations: AE, adverse event; CI, confidence interval; dTPA, diphtheria, tetanus, and acellular pertussis; dTpa_Placebo_RSV120, participants who received dTpa and placebo in the primary phase; GI, gastrointestinal; N, number of participants; RSV, respiratory syncytial virus; RSV60_dTpa_RSV120, participants who received RSV60 and dTpa in the primary phase; RSV60_Placebo_RSV120, participants who received RSV60 and placebo in the primary phase; RSV120_dTpa_RSV120, participants who received RSV120 and dTpa in the primary phase; RSV120_Placebo_RSV120, participants who received RSV120 and placebo in the primary phase.

Note: All solicited local (injection-site) reactions were considered causally related to vaccination, as per protocol.
