# Supplementary material for: Safety and Immunogenicity of Respiratory Syncytial Virus Prefusion Maternal Vaccine Coadministered With Diphtheria-Tetanus-Pertussis Vaccine: A Phase 2 Study
Source: J Infect Dis. 2023 Dec 22;230(2):e353–62. doi: 10.1093/infdis/jiad560 (PMC11326842; doi:10.1093/infdis/jiad560)
Supplement: jiad560_Supplementary_Data [file jiad560_supplementary_data.zip › Supplementary_Table_5.docx]

**Supplementary Table 5.** Most Common AEs Observed Within 30 Days of the Second Vaccination (Reported in ≥3% of Patients in any Treatment Group; Extension Phase) — Exposed Set

|  | RSV120_dTpa_RSV120  (n=39) | RSV120_Placebo_RSV120  (n=41) | RSV60_dTpa_RSV120  (n=46) | RSV60_Placebo_RSV120  (n=41) | dTpa_Placebo_RSV120  (n=44) |
| --- | --- | --- | --- | --- | --- |
| Headache, n (%) | 1 (3) | 1 (2) | 1 (2) | 3 (7) | 2 (4) |
| 95% CI | (0, 13) | (0, 13) | (0, 12) | (2, 20) | (1, 15) |
| Fatigue, n (%) | 1 (3) | 1 (2) | 1 (2) | 1 (2) | 0 |
| 95% CI | (0, 13) | (0, 13) | (0, 12) | (0, 13) | (0, 8) |
| Oropharyngeal pain, n (%) | 0 | 3 (7) | 1 (2) | 0 | 0 |
| 95% CI | (0, 9) | (2, 20) | (0, 12) | (0, 9) | (0, 8) |
| Injection-site pruritus, n (%) | 0 | 1 (2) | 0 | 2 (5) | 0 |
| 95% CI | (0, 9) | (0, 13) | (0, 8) | (1, 17) | (0, 8) |
| Malaise, n (%) | 1 (3) | 0 | 1 (2) | 0 | 0 |
| 95% CI | (0, 13) | (0, 9) | (0, 12) | (0, 9) | (0, 8) |
| Pharyngitis, n (%) | 0 | 0 | 0 | 2 (5) | 0 |
| 95% CI | (0, 9) | (0, 9) | (0, 8) | (1, 17) | (0, 8) |
| Limb discomfort, n (%) | 1 (3) | 0 | 0 | 0 | 1 (2) |
| 95% CI | (0, 13) | (0, 9) | (0, 8) | (0, 9) | (0, 12) |
| Administration-site pain, n (%) | 1 (3) | 0 | 0 | 0 | 0 |
| 95% CI | (0, 13) | (0, 9) | (0, 8) | (0, 9) | (0, 8) |
| Injection-site induration, n (%) | 1 (3) | 0 | 0 | 0 | 0 |
| 95% CI | (0, 13) | (0, 9) | (0, 8) | (0, 9) | (0, 8) |
| Abdominal pain, n (%) | 1 (3) | 0 | 0 | 0 | 0 |
| 95% CI | (0, 13) | (0, 9) | (0, 8) | (0, 9) | (0, 8) |
| Diarrhea, n (%) | 1 (3) | 0 | 0 | 0 | 0 |
| 95% CI | (0, 13) | (0, 9) | (0, 8) | (0, 9) | (0, 8) |
| Gastrointestinal disorder, n (%) | 1 (3) | 0 | 0 | 0 | 0 |
| 95% CI | (0, 13) | (0, 9) | (0, 8) | (0, 9) | (0, 8) |
| Vomiting, n (%) | 1 (3) | 0 | 0 | 0 | 0 |
| 95% CI | (0, 13) | (0, 9) | (0, 8) | (0, 9) | (0, 8) |
| Arthralgia, n (%) | 1 (3) | 0 | 0 | 0 | 0 |
| 95% CI | (0, 13) | (0, 9) | (0, 8) | (0, 9) | (0, 8) |

Abbreviations: AE, adverse event; CI, confidence interval; dTPA, diphtheria, tetanus, and acellular pertussis; dTpa_Placebo_RSV120, participants who received dTpa and placebo in the primary phase; N, number of participants; RSV, respiratory syncytial virus; RSV60_dTpa_RSV120, participants who received RSV60 and dTpa in the primary phase; RSV60_Placebo_RSV120, participants who received RSV60 and placebo in the primary phase; RSV120_dTpa_RSV120, participants who received RSV120 and dTpa in the primary phase; RSV120_Placebo_RSV120, participants who received RSV120 and placebo in the primary phase.

Note: All solicited local (injection-site) reactions were considered causally related to vaccination, as per protocol.
