# Supplementary material for: Safety and Immunogenicity of Respiratory Syncytial Virus Prefusion Maternal Vaccine Coadministered With Diphtheria-Tetanus-Pertussis Vaccine: A Phase 2 Study
Source: J Infect Dis. 2023 Dec 22;230(2):e353–62. doi: 10.1093/infdis/jiad560 (PMC11326842; doi:10.1093/infdis/jiad560)
Supplement: jiad560_Supplementary_Data [file jiad560_supplementary_data.zip › Supplementary_Table_6.docx]

**Supplementary Table 6.** Group Difference Between RSV120_dTpa and dTpa_Placebo, and Between RSV60_dTpa and dTpa_Placebo, in the Percentage of Participants with Seroprotection^a^ at Day 31 after Vaccination (Primary Phase) — Per-Protocol Set

| **Antibody** |  | **RSV120_dTpa** | **dTpa_Placebo** | **Between-group difference** |
| --- | --- | --- | --- | --- |
| Anti-D antibody concentration | N | 96 | 97 |  |
|  | n | 96 | 96 |  |
|  | % | 100.0 | 99.0 | 1.0 |
|  | 95% CI | 96.2, 100.0 | 94.4, 100.0 | –2.9, 5.6 |
| Anti-T antibody concentration | N | 98 | 97 |  |
|  | n | 98 | 97 |  |
|  | % | 100.0 | 100.0 | 0.0 |
|  | 95% CI | 96.3, 100.0 | 96.3, 100.0 | –3.8, 3.8 |
| **Antibody** |  | **RSV60_dTpa** | **dTpa_Placebo** | **Between-group difference** |
| Anti-D antibody concentration | N | 99 | 97 |  |
|  | n | 98 | 96 |  |
|  | % | 99.0 | 99.0 | 0.0 |
|  | 95% CI | 94.5, 100.0 | 94.4, 100.0 | –4.6, 4.7 |
| Anti-T antibody concentration | N | 102 | 97 |  |
|  | n | 102 | 97 |  |
|  | % | 100.0 | 100.0 | 0.0 |
|  | 95% CI | 96.4, 100.0 | 96.3, 100.0 | –3.6, 3.8 |

Abbreviations: 95% CI, two-sided standardized asymptotic 95% confidence interval; dTPA, diphtheria, tetanus, and acellular pertussis; dTpa_Placebo, participants who received dTpa and placebo; N, number of participants; n/%, number/percentage of participants in a given category; RSV, respiratory syncytial virus; RSV60_dTpa, participants who received RSV60 and dTpa; RSV120_dTpa, participants who received RSV120 and dTpa.

^a^Anti-diphtheria (D) or anti-tetanus (T) antibody concentrations ≥0.1 IU/mL by enzyme-linked immunosorbent assay.
