# Supplementary material for: Safety and Immunogenicity of Respiratory Syncytial Virus Prefusion Maternal Vaccine Coadministered With Diphtheria-Tetanus-Pertussis Vaccine: A Phase 2 Study
Source: J Infect Dis. 2023 Dec 22;230(2):e353–62. doi: 10.1093/infdis/jiad560 (PMC11326842; doi:10.1093/infdis/jiad560)
Supplement: jiad560_Supplementary_Data [file jiad560_supplementary_data.zip › Supplementary_Table_7.docx]

**Supplementary Table 7**. Number and Percentage of Subjects with RSV-A Neutralizing Antibody Titer Equal to or Above 18 ED60 and GMT and GMR (Extension Phase) – Per Protocol Set

|  | RSV120_dTpa | RSV120_Placebo | RSV60_dTpa | RSV60_Placebo | dTpa_Placebo |
| --- | --- | --- | --- | --- | --- |
| Participants with pre-second vaccination results, N | 38 | 40 | 46 | 41 | 46 |
| % of participants ≥18 years, % (95% CI) | 100 (90.7, 100) | 100 (91.2, 100) | 100 (92.3, 100) | 100 (91.4, 100) | 100 (92.3, 100) |
| ED60 GMT | 2027 (1546, 2656) | 2889 (2086, 4001) | 2885 (2204, 3778) | 2302 (1703, 3064) | 759 (588, 980) |
| Participants with results at Day 31 post-second vaccination, N | 38 | 37 | 44 | 39 | 42 |
| % of participants ≥18 years, % (95% CI) | 100 (90.7, 100) | 100 (90.5, 100) | 100 (92.0, 100) | 100 (91.0, 100) | 100 (91.6, 100) |
| ED60 GMT | 3892 (3016, 5022) | 5071 (3848, 6683) | 4779 (3747, 6094) | 4920 (3886, 6231) | 8200 (6380, 10539) |
| Visit comparison/baseline (pre-second vaccination) | | | | | |
| Participants with results pre- and post-second vaccination, N | 38 | 31 | 44 | 39 | 42 |
| GMR (95% CI) | 1.92 (1.52, 2.43) | 1.91 (1.47, 2.49) | 1.72 (1.47, 2.02) | 2.07 (1.63, 2.62) | 11.13 (9.30, 14.92) |

Abbreviations: CI, confidence interval; dTPA, diphtheria, tetanus, and acellular pertussis; dTpa_Placebo, participants who received dTpa and placebo; ED60, estimated dilution 60; GMR, geometric mean of individual ratio of antibody titers at each post-vaccination timepoint over pre-vaccination; GMT, geometric mean antibody titer calculated on all subjects; RSV, respiratory syncytial virus; RSV60_dTpa, participants who received RSV60 and dTpa; RSV60_Placebo, participants who received RSV60 and placebo; RSV120_dTpa, participants who received RSV120 and dTpa; RSV120_Placebo, participants who received RSV120 and placebo.
